# Real-Time Weighted Data Fusion Algorithm for Temperature Detection Based on Small-Range Sensor Network

**DOI:** 10.3390/s19010064

**Published:** 2018-12-25

**Authors:** Ziling Zhang, Xinyuan Nan, Cong Wang

**Affiliations:** College of electrical Engineering, Xinjiang University, Urumqi 830047, China; xynan@xju.edu.cn (X.N.); wangcong1120@foximail.com (C.W.)

**Keywords:** distributed sensor fusion, small-range sensor network, iterative operation, multi-fading factor, weighted fading memory index

## Abstract

Biological oxidation pretreatment, which can improve the yield of gold, is the main gold extraction technology for disposing refractory gold ore with high arsenic and sulfur. The temperature of the oxidation tank influences the oxidation efficiency between the ore pulp and bacteria, including the yield of gold. Therefore, measurement has consistently been an important subject for researchers. As an effective data processing method, data fusion has been used extensively in many fields of industrial production. However, the interference of equipment or external factors such as the diurnal temperature difference or powerful wind may constantly increase measurement errors and damage certain sensors, which may transmit error data. These problems can be solved by following a pretreatment process. First, we establish a heat transfer mechanism model. Second, we design a small-range sensor network for the pretreatment process and present a layered fusion structure of sharing sensors using a multi-connected fusion structure. Third, we introduce the idea of iterative operation in data processing. In addition, we use prior data for predicting state values twice in order to improve the effectiveness of extended Kalman filtering in one time step. This study also proposes multi-fading factors on the basis of a weighted fading memory index to adjust the prediction error covariance. Finally, the state estimation accuracy of each sensor can be used as a weighting principle for the predictive confidence of each sensor by adding a weighting factor. In this study, the performance of the proposed method is verified by simulation and compared with the traditional single-sensor method. Actual industrial measurement data are processed by the proposed method for the equipment experiment. The performance index of the simulation and the experiment shows that the proposed method has a higher global accuracy than the traditional single-sensor method. Simulation results show that the accuracy of the proposed method has a 55% improvement upon that of the traditional single-sensor method, on average. In the equipment experiment, the accuracy of the industrial measurement improved by 37% when using the proposed method.

## 1. Introduction

**Figure 1:**
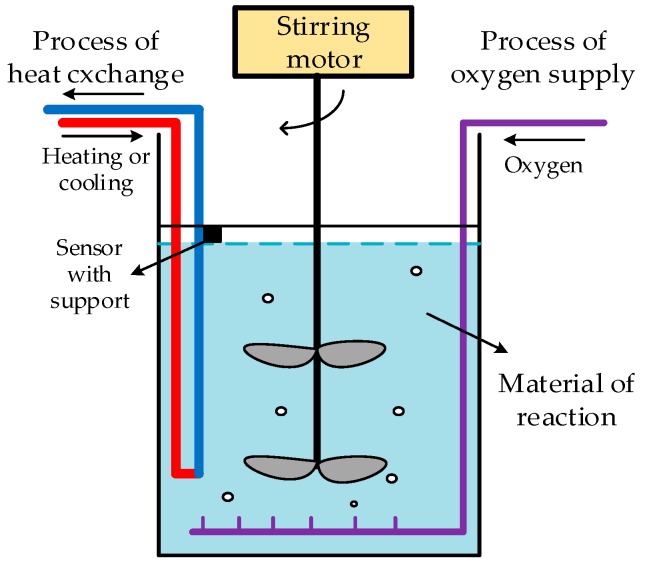
Schematic of biological oxidation pretreatment.

Data fusion has been widely used in military and civilian applications. Biological oxidation pretreatment is a novel but difficult strategy of industrial gold production [1]. The process of biological oxidation pretreatment uses bacteria that oxidize the mineral wrapped with gold, and expose the gold outside. The control process has several important factors, such as temperature, oxygen quantity, pH value, and ORP (oxidation-reduction potential). To ensure the activity of bacteria, the temperature accuracy of the measurement and control processes becomes important. Figure 1 shows the biological oxidation pretreatment:

As an effect method, data fusion was first applied to the military field, which attracted the attention of researchers. Then, data fusion became widely used in many other fields. For example, Skladnev built a probabilistic model to simulate 90 to 100 patients’ measurements, and used data fusion to assess the patients’ conditions. The simulated addition of Hypo Mon data produced an improvement in the CGMS (continuous glucose monitoring system) hypoglycemia alarm performance of 10% at equal specificity [2]. Seal used three types of data fusion ranking algorithms on the PknB dataset, namely: sum rank, sum score, and reciprocal rank to select compounds in a virtual screening process and determine the best approach [3]. Rigatos proposed extended and unscented information filters for the condition monitoring of electric power transmission and distribution systems [4]. Maschi proposed a method for collecting and processing local data through a data fusion technology. The method effectively reduced the volume of data generated and decreased the volume of messages generated by the Internet of Things environment [5]. Moussavi Khalkhali introduced Dempster–Shafer evidential theory and proportional conflict redistribution rule number five into intelligent housing systems to solve the problem of room security [6]. Tsinganos compared three newly proposed data fusion schemes that have been applied in human activity recognition and fall detection for old people. The author proposed a machine learning strategy that provided a useful inspiration for the study of drop detection [7]. Pan proposed a gray Kalman filter and a novel open-circuit voltage model for the estimation of the state of charge of lithium-ion batteries to improve the accuracy of charge estimation [8]. As an important branch of data fusion, multi-sensor data fusion has also been used in industrial processes [9,10,11,12].

**Figure 2:**
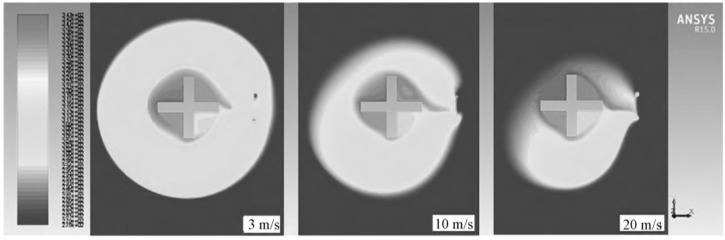
Temperature distribution of temperature in different wind field positions.

The present work aims to solve the problems related to the detection and estimation of temperature. In previous studies on temperature treatment, Zhao designed an ultra-low temperature online monitoring system and introduced the correlation function of a careless error elimination method on the basis of fuzzy theory. This method improves the accuracy of sensor data [13] and obtains a good effect. Regarding the problems of temperature and humidity detection in an industrial environment, Lv used a fuzzy set-based approach for multi-sensor data related to temperature and humidity fusion. The approach solves the problem of inaccurate data caused by a large industrial range [14]. The author also implemented the idea in hardware. Sung used adaptive fuzzy logic algorithms to compute the monitoring area temperature in a 30 m (L) × 40 m (W) × 15 m (H) room [15]. The authors in [16,17] used the Bayesian estimation combined with an improved array of sensors to solve the problem of high-precision measurement by non-contact sensors and performance by boiler heat treatment. In a plant factory, Cui eliminated the gross error by using the Dixon criterion, and then used adaptive weighting data fusion to obtain an accurate temperature value [18]. The results have greatly inspired and helped the current study. Such results also provide guidance for temperature processing that uses data fusion. However, the aforementioned studies mainly focused on an ideal external environment situation, for example, in a room, or a thermostatic chamber, or a high-temperature environment [17], which is not easily disturbed by the outside world. In [14], the author conducted research in an indoor environment to solve the problem of multi-field fusion in different industrial sites. The research object of biological oxidation pretreatment in the present study is different from those of previous studies for the following reasons. First, due to the characteristics of minerals that contain sulfur, arsenic, and other substances, the location of the factory is consistently in remote areas. Second, the reactor of biological oxidation pretreatment is set on an outdoor field. Third [19] analyzed the problem of the “sensor actuator effect” in greenhouses. The sensor near the temperature regulator was considerably affected due to the placement of the temperature regulator; however, the sensor that was far from the temperature regulator could not evidently change. Therefore, temperature detection was inaccurate, and the current study obtains the same result. Last but not least is that at present, the common schemes in actual industrial sites are using single sensor measurement. In order to save the cost of building a reactor, most schemes place the single sensor on the edge of the reactor. However, the environment of the object in this paper is very harsh. In the former research, we have analyzed the temperature distribution of the reactor in detail. In [20], Figure 2 shows the cloud distribution of the temperature of an oxidation tank in a wind field in different positions. As we can see, when a sensor is placed in an ideal position, we can obtain a measurement with high accuracy. However, the temperature field is very uneven. Some regions can’t reach the setting value, thereby reducing the industrial yield.

To improve the global accuracy of temperature detection, this study presents a distributed sensor fusion algorithm for severe environments.

(1) The mechanism of heat transfer in biological oxidation pretreatment is analyzed, and a suitable state model is also established to facilitate prediction and measurement.

(2) We define a structure of data fusion on the basis of a multi-connected fusion structure. The sensors in the network are divided into three levels, in which each sensor can measure the temperature and process data. The level of sensors is determined by their location in the reactor. Low-level sensors play an important role in global estimation and accuracy improvement.

(3) Regarding data processing, we improve the traditional extended Kalman filtering (EKF). First, we introduce the idea of iterative operation for the state prediction. Then, multi-fading factors are improved by using a weighted fading memory index.

(4) In comparison with the traditional single-sensor measurement method, we propose a small-range sensor fusion method. The proposed method can fully consider the influence of external factors on the reactor temperature.

The remainder of this paper is organized as follows. Section 2 elaborates the algorithm, including the model of heat transfer in an oxidation tank, the improvement of the traditional EKF, and the criterion of the distributed sensor fusion. Section 3 presents the simulation and experiment to demonstrate the performance of the proposed algorithm. Section 4 draws the conclusions, and discusses future work with corresponding solutions in this study.

## 2. Algorithm Description

### 2.1. Heat Transfer Model of Oxidation Tank

When heat is transferred between a tube and a gold pulp, heat transfer obeys the Fourier convection heat transfer law, which is shown as follows:

(1) $$-\delta_{\tau }\frac{\partial \tau_{h}}{\partial f}=h(\tau_{h}-\tau_{o})$$

where $\delta_{\tau }$ represents the heat conductivity of snake-shaped casing and ore pulp, $\tau_{h}$ is the temperature of the snake-shaped casing, $\tau_{o}$ is the ore pulp temperature, h is the coefficient of heat transfer, and f is a contact point between the ore pulp and snake-shaped casing. The heat exchange of ore pulp obeys the law of the conservation of energy, which is shown as follows:

(2) $$\rho C(\frac{\partial \tau }{\partial t}+v_{x}\cdot \frac{\partial \tau }{\partial x}+v_{y}\cdot \frac{\partial \tau }{\partial y}+v_{z}\cdot \frac{\partial \tau }{\partial z})=\delta_{\tau }(\frac{\partial^{2}\tau }{\partial x^{2}}+\frac{\partial^{2}\tau }{\partial y^{2}}+\frac{\partial^{2}\tau }{\partial z^{2}})+\varsigma +w$$

where ρ is the pulp density; C is the specific heat capacity, t∈[0,T]; and $v_{x}$ , $v_{y}$, and $v_{z}$ represent the heat transfer velocity in the x , y, and z directions, respectively. τ is the measurement of temperature; ς is the temperature of heat source, which is the tube temperature; and w is the white noise of temperature, ‖w‖<<‖ς‖. Given the effect of wind and other factors, the heat of gold pulp is transferred to the environment through the reactor wall, which can be expressed as follows:

(3) $$q=\delta_{r}(\tau_{o}-\tau_{r})$$

where $\tau_{o}$ is the ore pulp temperature, $\tau_{r}$ is the reactor wall temperature, and $\delta_{r}$ represents the heat conductivity of the reactor wall and ore pulp. Therefore, the discrete equation of heat exchange in gold pulp is:

(4) $$X_{k}=\left[\begin{array}{l} \tau_{k}\\ \varsigma_{k} \end{array}\right]=\left[\begin{array}{cc} F_{k-1} & \Delta t\cdot I\\ 0 & F_{k-1}^{,} \end{array}\right]\left[\begin{array}{l} \tau_{k-1}\\ \varsigma_{k-1} \end{array}\right]+\left[\begin{array}{l} w_{k}\\ 0 \end{array}\right]$$

where $\tau_{k},\varsigma_{k},w_{k}\in R^{n}$, and Δt is the time step. In each time step, sensors are used to measure the reactor temperature. The measurement equation can be expressed as follows:

(5) $$Z_{k}=H_{k}^{T}\tau_{k}+v_{k}$$

where $H_{k}\in R^{n\times m}$ , and $v_{k}$ is the white noise of measurement.

### 2.2. Fusion System of Biological Oxidation Pretreatment

Comparison between a single sensor and multi-sensors reveals that the latter provides more abundant sources of information [21,22], whether in the geographical region or spectrum range. Given several measurements, multi-sensor data fusion can improve the performance of detection and reduce the rate of false alarm.

Measurements of multi-sensors can be fused in a central site or many sites. The fusion structure of centralized data fusion must transmit all of the information from single sensors to the central site. All of the measurements are guaranteed theoretically without loss [23]; however, this method increases the computational intensity of the central site, and is vulnerable to gross errors. Conversely, distributed data fusion requires a low communication bandwidth, because each sensor transmits processed data instead of raw data [4,24,25]. Thus, distributed data fusion has been used in numerous situations.

**Figure 3:**
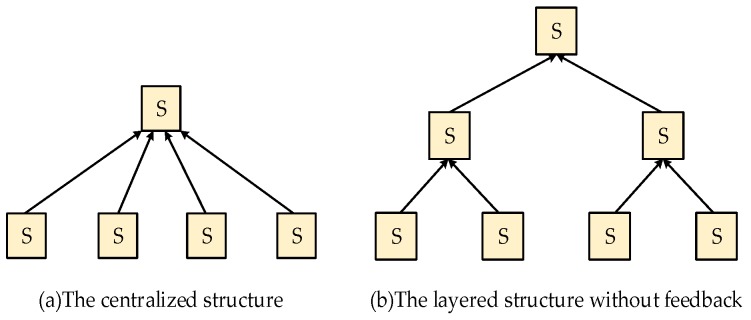
Single-connected fusion structures.

**Figure 4:**
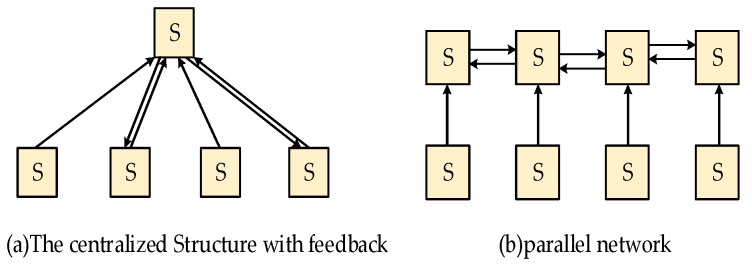
Multi-connected fusion structures.

Before we establish the array of the sensor network, the appropriate fusion structure of distributed data fusion must be determined. On the basis of the number of communication paths from one sensor to another, distributed data fusion can be divided into a single-connected and multi-connected fusion structures. In the single-connected fusion structure, each sensor is connected by only one path, whereas for a multi-connected fusion structure, sensors can be connected by more than one path. Figure 3 and Figure 4 show the several types of single-connected and multi-connected fusion structures, respectively.

**Figure 5:**
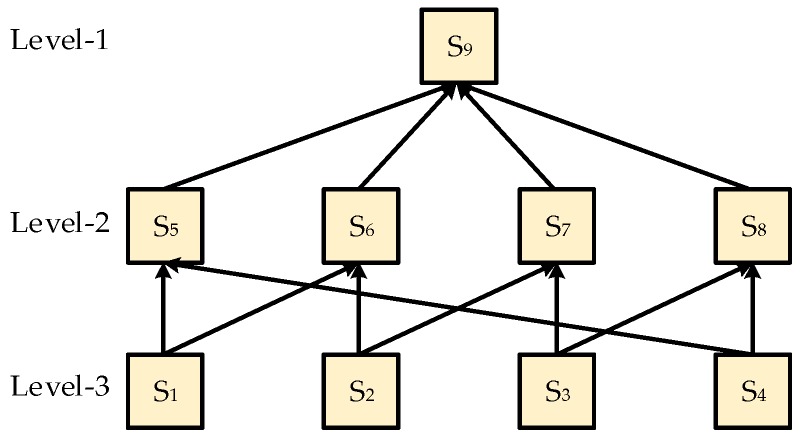
Layered fusion structure of sharing sensors.

In traditional temperature measurement, sensors are placed in certain positions on the top of the reactor and are in contact with the material of reaction. This traditional measurement has considerable limitations. That is, the method cannot detect the changes at the edge of the reactor, thereby leading to local inaccuracies in the temperature of different regions, and the result of measurement temperature is accurate only in the surrounding of the sensor. Moreover, other areas of the reactor may not meet the temperature setting, thereby causing a low production quality. Therefore, we propose a new layered fusion structure of sharing sensors on the basis of a multi-connected fusion structure. This new structure consists of nine sensors, which are divided into three levels, as shown in Figure 5.

Sensors one to four represent level-three sensors, which are the lowest-level sensors. We use them to measure the temperature in the most disturbed areas of the reactor. The data measured by level-three sensors represent the global variation trend of the reactor temperature. As described in Section 1, level-three sensors can be fused with level-two sensors alternately by consulting the loop structure of the reactor. After fusing with level-three sensors, level-two sensors consisting of sensors five to eight are then fused with level-one sensor nine to modify the final measurement. 

**Figure 6:**
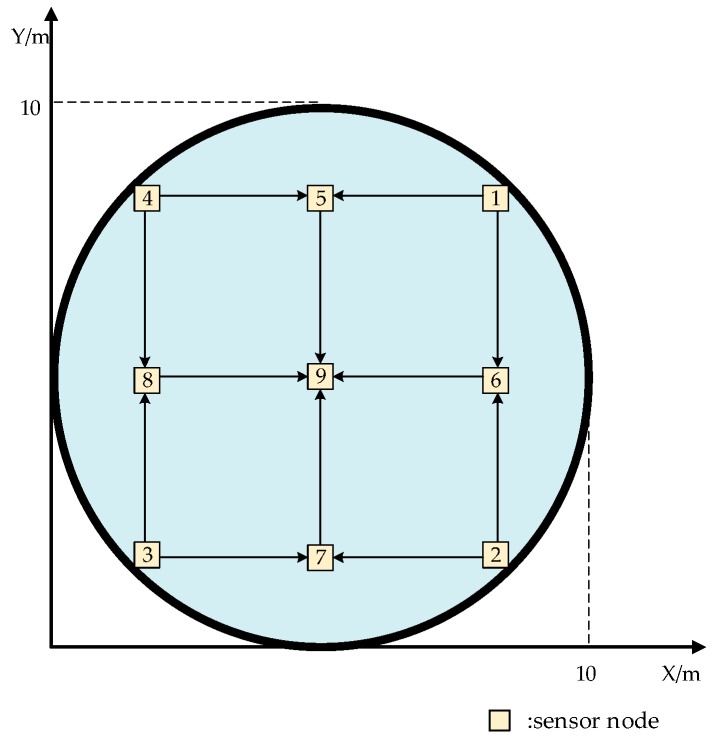
Sensor array of temperature measurement.

The small-range sensor network can be proposed on the basis of the fusion structure, as shown in Figure 6.

### 2.3. Improvements of Data Processing

#### 2.3.1. Iterative Operation of State Model

EKF is widely used in distributed sensor fusion. The traditional EKF method is based on the Taylor expansion. A linear function is used to approximate the nonlinear function, and the state estimation of the nonlinear system is then obtained. As described in Section 2.1, consider that a sensor node of the network takes the following observation:

(6) $$X_{k+1}=F_{k}(X_{k},W_{k})$$

(7) $$Z_{k}=H_{k}(X_{k},V_{k})$$

where X denotes the reactor temperature state vector; and $W_{k}$ and $V_{k}$ represent the system state noise and observation noise, respectively. Assume that $W_{k}$ and $V_{k}$ are Gaussian white noise with zero mean, and $Q_{k}$ and $R_{k}$ are covariance matrixes, where $Q_{k}$ is a nonnegative definite matrix, and $R_{k}$ is a positive definite matrix. The state transfer function and the measurement function of the system are represented by the following expressions:

(8) $$F_{k}(\cdot )=\left[F_{k}^{1}(\cdot ),F_{k}^{2}(\cdot ),\cdots ,F_{k}^{n}(\cdot )\right]$$

(9) $$H_{k}\left(\cdot \right)=\left[H_{k}^{1}\left(\cdot \right),H_{k}^{2}\left(\cdot \right),\cdots ,H_{k}^{m}\left(\cdot \right)\right]$$

Assume that the state estimation of the system and the error covariance are ${\hat{X}}_{k|k}$ and $P_{k|k}$ , respectively. Then, the first-order Taylor expansion is used to linearize the state function, which can be expressed as follows:

(10) $$X_{k+1}=F_{k}(X_{k},W_{k})\approx F_{k}({\hat{X}}_{k|k},0)+F_{k}^{X}{\tilde{X}}_{k|k}+F_{k}^{W}W_{k}$$

where ${\tilde{X}}_{k|k}=X_{k}-{\hat{X}}_{k|k}$.

The error covariance prediction at the *k* + 1 moment is:

(11) $$P_{k+1|k}=F_{k}^{X}{\hat{X}}_{k|k}{\hat{X}}_{k|k}^{T}{\left(F_{k}^{X}\right)}^{T}+F_{k}^{W}W_{k}W_{k}^{T}{\left(F_{k}^{W}\right)}^{T}=F_{k}^{X}P_{k|k}{\left(F_{k}^{X}\right)}^{T}+F_{k}^{W}Q_{k}{\left(F_{k}^{W}\right)}^{T}$$

The gain matrix can be defined as:

(12) $$K_{k+1}=R_{{\tilde{Z}}_{k+1|k}{\tilde{X}}_{k+1|k}}{\left(R_{{\tilde{Z}}_{k+1|k}{\tilde{Z}}_{k+1|k}}\right)}^{-1}\approx P_{k+1|k}{\left(H_{k+1}^{X}\right)}^{T}{\left[H_{k+1}^{X}P_{k+1|k}{\left(H_{k+1}^{X}\right)}^{T}+H_{k+1}^{V}R_{k+1}{\left(H_{k+1}^{V}\right)}^{T}\right]}^{-1}$$

Moreover, the update measurement of EKF is:

(13) $${\hat{X}}_{k+1|k+1}={\hat{X}}_{k+1|k}+K_{k+1}Z_{k+1}-H_{k+1}^{X}{\hat{X}}_{k+1|k}$$

(14) $$P_{k+1|k+1}=P_{k+1|k}-K_{k+1|k}H_{k+1|k}P_{k+1|k}$$

In [8,26,27,28], distributed data fusion was introduced on the basis of EKF to solve different projects. However, how the EKF uses the first-order Taylor expansion to linearize the system yields the error of the high-order part. In a system with strong nonlinearity, the effect of error is serious. The error of each single sensor can reduce the accuracy of the fusion result. To solve this problem, we introduce the idea of iterative operation. In the process of measurement updates in EKF, we obtain ${\hat{X}}_{k+1|k+1}$ and $P_{k+1|k+1}$, and use them to replace ${\hat{X}}_{k+1|k}$ and $P_{k+1|k}$ , as shown in Equations (15) and (16):

(15) $${\left(F_{k+1}^{X}\right)}^{\left(j\right)}={\frac{\partial F_{k+1}(X_{k+1},0)}{\partial X_{k+1}}|}_{X_{k+1}={\hat{X}}_{k+1|k+1}}^{\left(j\right)}$$

(16) $${\left(F_{k+1}^{W}\right)}^{\left(j\right)}={\frac{\partial F_{k+1}({\hat{X}}_{k+1|k+1},W_{k+1})}{\partial W_{k+1}}|}_{W_{k+1}=0}^{\left(j\right)}$$

where ${\left(F_{k+1}^{X}\right)}^{\left(j\right)}$ and ${\left(F_{k+1}^{W}\right)}^{\left(j\right)}$ represent another expansion of the state function, and the iterative operation times is j. The corresponding iterative covariance matrix may be expressed as follows:

(17) $$P_{k+1|k}^{\left(j\right)}={\left(F_{k+1}^{X}\right)}^{\left(j\right)}P_{k+1|k}{\left[{\left(F_{k+1}^{X}\right)}^{\left(j\right)}\right]}^{T}+{\left(F_{k+1}^{W}\right)}^{\left(j\right)}Q_{k+1}{\left[{\left(F_{k+1}^{W}\right)}^{\left(j\right)}\right]}^{T}$$

Therefore, the updated measurement can be described as follows:

(18) $$K_{k+1|k}^{\left(j\right)}=P_{k+1|k}^{\left(j\right)}{\left(H_{k+1}^{X}\right)}^{T}{\left[H_{k+1}^{X}P_{k+1|k}^{\left(j\right)}{\left(H_{k+1}^{X}\right)}^{T}+H_{k+1}^{V}R_{k+1}{\left(H_{k+1}^{V}\right)}^{T}\right]}^{-1}$$

(19) $${\hat{X}}_{k+1|k}^{\left(j\right)}=F_{k+1}({\hat{X}}_{k+1|k+1},W_{k})$$

(20) $${\hat{X}}_{k+1|k+1}^{\left(j\right)}={\hat{X}}_{k+1|k}^{\left(j\right)}+K_{k+1|k}^{\left(j\right)}\cdot (Z_{k+1}-H_{k+1}^{X}{\hat{X}}_{k+1|k}^{\left(j\right)})$$

(21) $$P_{k+1|k+1}^{\left(j\right)}=P_{k+1|k}^{\left(j\right)}-K_{k+1|k}^{\left(j\right)}H_{k+1}^{X}P_{k+1|k}^{\left(j\right)}$$

#### 2.3.2. Multiple Fading Factors Based on Weighted Fading Memory Index

EKF has self-memory [29]; thus, the results processed by EKF depend on the sensor data in each moment. Given the divergence caused by the error of temperature measurement, previous data can play a restraining role in the process. The correction of the next prediction value by the information matrix obtained from the new data will be restrained. Therefore, the filter value cannot track the true value well. In a previous study, a fading factor was added into the error covariance matrix [30]. However, using a single fading factor cannot sufficiently correct the multi-variable error covariance matrix. Therefore, the method of a single fading factor must be improved. In the current study, the multi-fading factors based on a weighted fading memory index are used to adjust the prediction error covariance. In Equation (11), the multi-fading factors that are added into the prior error covariance are as follows:

(22) $$\lambda_{k+1}=diag\{\lambda_{k+1}(1),\lambda_{k+1}(2)\cdots \lambda_{k+1}(n)\}$$

where the representation of the innovation matrix (residual matrix) is as follows:

(23) $${\hat{D}}_{0,k+1}=\frac{1}{k}\sum_{i=1}^{k+1}\gamma_{i}\gamma_{i}^{T}$$

where $\gamma_{i}$ is the innovation vector at time i=1⋯k.

As a result, we obtain the following:

(24) $$\{\begin{array}{l} C_{k+1}={\overset{\wedge }{D}}_{o,k+1}-H_{k+1}^{X}F_{k}^{W}Q_{k}{\left(F_{k}^{W}\right)}^{T}{\left(H_{k+1}^{X}\right)}^{T}-R_{k+1}\\ G_{k+1}=(H_{K+1}(\cdot ))J_{k+1}{\left(H_{K+1}(\cdot )\right)}^{T}\\ J_{k+1}=F_{k}^{X}P_{k|k}{\left(F_{k}^{X}\right)}^{T} \end{array}$$

We can also obtain:

(25) $$\lambda_{k+1}G_{k+1}=C_{k+1}$$

Thus, the multi-fading factors at moment *k* + 1 can be expressed as follows:

(26) $$\lambda_{k+1}(i)=\left\{ \begin{array}{l} \max\left\{1,\frac{C_{k+1}(i,i)}{H_{k+1}^{i}(\cdot )J_{k+1}(i,i)}\right\},i=1,2,\ldots ,m\\ 1,i=m+1,m+2,\ldots ,n \end{array}\right.$$

Therefore, Equation (15) can be expressed as:

(27) $$P_{k+1|k}=\lambda_{k+1}F_{k}^{X}P_{k|k}{\left(F_{k}^{X}\right)}^{T}+F_{k}^{W}Q_{k}{\left(F_{k}^{W}\right)}^{T}$$

In Equation (26), the calculation of multiple factors depends on the estimation of the innovation vector, which is expressed in Equation (23). The general calculation method in Equation (23) is used to determine the value of ${\hat{D}}_{0,k+1}$ by the arithmetic averaging of the historical data, which cannot raise the weight of the covariance matrix of the innovation vector. To emphasize the role of the recent innovation series and reduce the effect of old data, this study presents a novel covariance estimation method based on a weighted fading memory index. Unlike the general calculation in Equation (23), which uses the average weighting technique, such as 1/*k*, $\beta_{i}$ is introduced as the weight of the innovation vector matrix at different times. The new data weighting coefficient is large, whereas the old data weighting coefficient is small.

(28) $$\sum_{i=1}^{k}\beta_{i}=1,\beta_{i-1}=\beta_{i}b,0<b<1$$

where b is the forgetting factor that usually selects values between 0.7 and 0.95 because of the following formulas:

(29) $$\{\begin{array}{c} b^{0}+b^{1}+\ldots +b^{k}=\frac{1-b^{k+1}}{1-b}\\ (b^{0}+b^{1}+\ldots +b^{k})\frac{1-b}{1-b^{k+1}}=1 \end{array}$$

Define $d_{k+1}=\frac{1-b}{1-b^{k+1}}$. Then, we can obtain $\beta_{i}=d_{k}\cdot b^{k-i}$ and use $\beta_{i}$ to replace 1/*k* in Equation (23).

(30) $${\hat{D}}_{0,k+1}=\sum_{i=1}^{k+1}\beta_{i}\gamma_{i}\gamma_{i}^{T}=\sum_{i=1}^{k+1}d_{k}\cdot b^{k-i}\gamma_{i}\gamma_{i}^{T}=d_{k}\gamma_{k}\gamma_{k}^{T}+d_{k}\sum_{i=1}^{k}\cdot b^{k-i}\gamma_{i}\gamma_{i}^{T}=d_{k}\gamma_{k}\gamma_{k}^{T}+\frac{d_{k}b}{d_{k-1}}d_{k-1}\sum_{i=1}^{k}\cdot b^{k-1-i}\gamma_{i}\gamma_{i}^{T}$$

(31) $$\frac{d_{k}b}{d_{k-1}}=\frac{\frac{1-b}{1-b^{k+1}}\cdot b}{\frac{1-b}{1-b^{k}}}=\frac{b-b^{k}}{1-b^{k}}=1-\frac{1-b}{1-b^{k}}=1-d_{k}$$

Therefore, the covariance matrix of the innovation vector can be expressed as follows:

(32) $${\hat{D}}_{0,k+1}=d_{k}\gamma_{k}\gamma_{k}^{T}+(1-d_{k})d_{k-1}\sum_{i=1}^{k}\cdot b^{k-1-i}\gamma_{i}\gamma_{i}^{T}$$

**Figure 7:**
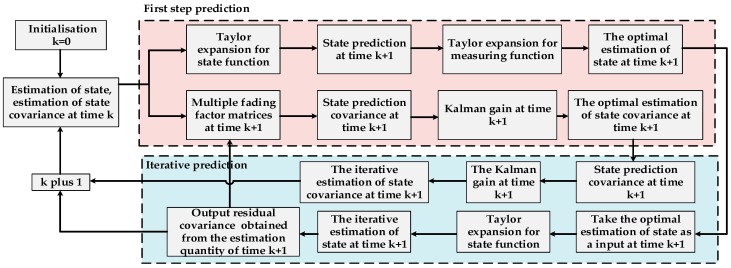
Procedure of improved extended Kalman filtering (IEKF).

The novel covariance estimation method based on a weighted fading memory index is simple. Computers can easily realize this method, and the value mainly depends on the selection of factor b. For the biological oxidation pretreatment system, which has violent interference, small factors are selected to ensure the availability of innovation vectors. Figure 7 shows the procedure of improved extended Kalman filtering.

### 2.4. Distributed Data Fusion Method Based on Improved EKF

Section 2.3 has introduced the data processing method in a sensor node. In this section, the distributed data fusion algorithm based on the fusion structure in Section 2.1 will be proposed. Define $\Psi_{k+1,i}$ as the local fusion value of sensor i with its corresponding low-level sensors. In addition, $N_{i}$ represents the set of sensor i with its corresponding low-level sensors. The processing data are assigned as the initial value of$\Psi_{k+1,i}$ , which combines with Equations (20) and (21), and is shown as follows:

(33) $$\Psi_{k+1,i}={\hat{X}}_{k+1|k+1,i}^{\left(j\right)};PP_{k+1,i}=P_{k+1|k+1,i}^{\left(j\right)}$$

The data are fused as follows:

For $1\in N_{i}$:

(34) $$\Psi_{k+1,i}=\Psi_{k+1,i}+K_{k+1|k,l}^{\left(j\right)}[Z_{k+1,l}-H_{k+1,l}^{X}\Psi_{k+1,i}]$$

(35) $$K_{k+1|k,l}^{\left(j\right)}=PP_{k+1,i}{\left(H_{k+1,l}^{X}\right)}^{T}\cdot {\left[H_{k+1,l}^{X}PP_{k+1,i}{\left(H_{k+1,l}^{X}\right)}^{T}+H_{k+1,l}^{V}R_{k+1,l}{\left(H_{k+1,l}^{V}\right)}^{T}\right]}^{-1}$$

(36) $$PP_{k+1,i}=PP_{k+1,i}-K_{k+1|k,l}^{\left(j\right)}H_{k+1}^{X}PP_{k+1,i}$$

End

The final fusion value is:

(37) $${\hat{X}}_{k+1|k+1,i}^{\left(j\right)}=\sum_{l\in N_{i}}\omega_{l}\Psi_{k+1,i}$$

(38) $$P_{k+1|k+1,i}^{\left(j\right)}=PP_{k+1,i}$$

We use the state estimation accuracy of each sensor as a weighting principle, and $\omega_{l}$ represents the weights in local fusion. Assume l as the number of sensors in the local fusion. $\sigma_{1}^{2},\sigma_{2}^{2},\ldots ,\sigma_{l}^{2}$ represents the mean square deviation of the sensor. $X_{k+1}$ is the true value of the state function. They also meet the following relations:

(39) $$\left\{ \begin{array}{l} {\hat{X}}_{k+1|k+1,i}^{\left(j\right)}=\sum_{l\in N_{i}}\omega_{l}\Psi_{k+1,i}\\ \sum_{l\in N_{i}}\omega_{l}=1 \end{array}\right.$$

In each time of local fusion, the total mean square deviation is:

(40) $$\sigma^{2}=E{\left[\sum_{l\in N_{i}}\omega_{l}(X_{k+1}-{\hat{X}}_{k+1|k+1,i}^{\left(1\right)}\right)}^{2}]=\sum_{l\in N_{i}}\omega_{l}\sigma_{l}^{2}$$

Equations (39) and (40) confirm a multivariate quadratic function that must have a minimum value. On the basis of the extreme value theory of multivariate function, the minimum value can be expressed as follows:

(41) $$\omega_{\min,i}=\frac{1}{\sigma_{i}^{2}\sum_{l\in N_{i}}\frac{1}{\sigma_{l}^{2}}}$$

The corresponding standard deviation of the sensor is as follows:

(42) $$\sigma_{\min}^{2}=\frac{1}{\sum_{l\in N_{i}}\frac{1}{\sigma_{l}^{2}}}$$

**Figure 8:**
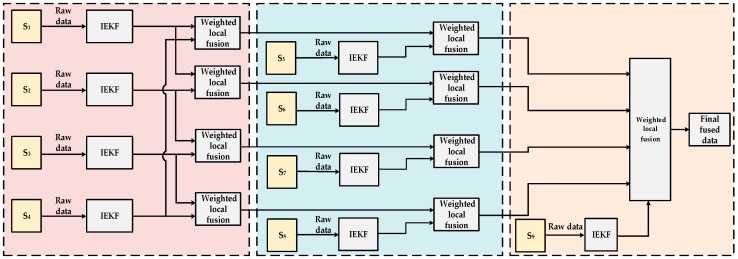
Distributed multi-sensor data fusion.

Through the weighted fusion of sensors of all levels, the temperature values processed by the distributed data fusion method are obtained. Figure 8 shows the entire process of the proposed method.

## 3. Simulation and Experiment

### 3.1. Simulation Results and Discussion

A simulation implemented in MATLAB is performed on a PC with a 2.5-GHz Intel Core CPU and 4 GB of memory to verify the effect of the improved distributed multi-sensor data fusion algorithm for the temperature measurement of the biological oxidation pretreatment process. In this simulation, the sensor nodes are located on the top of a 10-m diameter reactor and follow the array described in Section 2.2. The simulation model is set following the heat transfer model of the oxidation reactor. The object is the same for each sensor; thus, the PDF (probability density function) setting of object is coincident. However, given the different positions and levels of the sensors, the interference of the simulation is different. Therefore, the noise covariance settings for each sensor are different during the simulation. The simulation data of each sensor are recorded, for the reason that each sensor has different accuracies because of the influence of external factors and its own characteristics. Each sensor’s performance will be evaluated by using the mean absolute error (MAE), mean relative error (MRE), and root mean square error (RMSE). The calculation of these performances can be expressed as follows:

(43) $$MAE=\frac{1}{n}\sum_{k=1}^{n}\left|{\hat{X}}_{k+1|k+1}^{\left(j\right)}-X_{k+1}\right|$$

(44) $$MRE=\frac{1}{n}\sum_{k=1}^{n}\frac{\left|{\hat{X}}_{k+1|k+1}^{\left(j\right)}-X_{k+1}\right|}{X_{k+1}}$$

(45) $$RMSE=\sqrt{\frac{1}{n}{\sum_{k=1}^{n}\left({\hat{X}}_{k+1|k+1}^{\left(j\right)}-X_{k+1}\right)}^{2}}$$

where ${\hat{X}}_{k+1|k+1}^{\left(j\right)}$ represents the predicted value of each sensor, and $X_{k+1}$ represents the true value. 

The value of the single-sensor simulation is used as a contrast method of the algorithm. The location of the single sensor is the same as the position of level-one sensor nine. In each simulation, the proposed and contrast methods perform under the same conditions to ensure the consistency of the simulation. 

**Figure 9:**
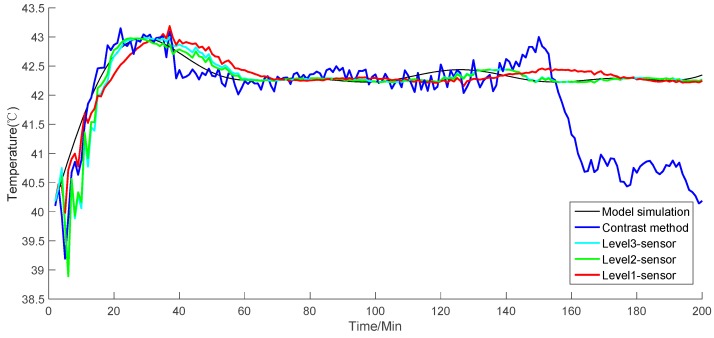
Simulation results of each level of the sensors and the contrast method.

**Figure 10:**
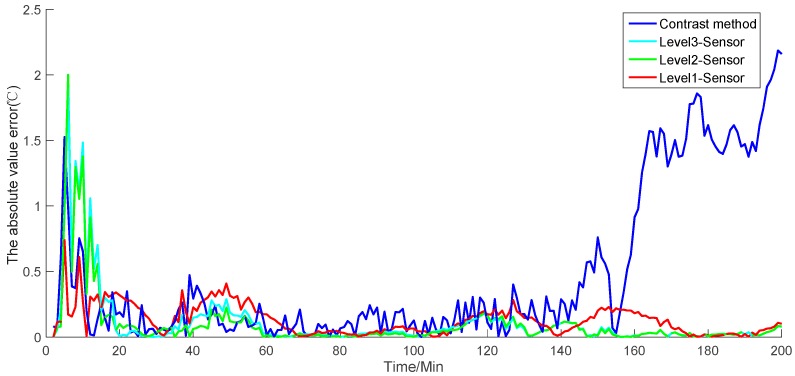
Absolute error of each level of the sensors and the contrast method.

**Figure 11:**
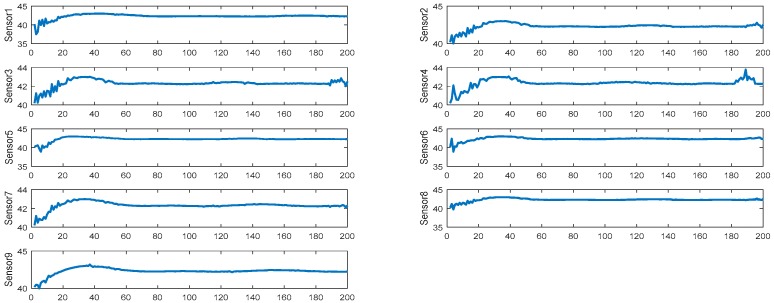
Simulation results of each sensor.

The simulation result in Figure 9 shows the value of the reactor temperature in the model simulation, the measurement of the contrast method, and the fusion value for each level of the sensors. Figure 10 shows the absolute value error of each simulation result, including the model simulation value of the temperature, the fusion value for each level of the sensors, and the simulation of the contrast method. Figure 11 presents the recorded simulation of each sensor. In Figure 9 and Figure 10, each level of the sensors shows the average value of all of the sensors.

The simulation results show that the contrast method has a strong fluctuation (Figure 9 and Figure 10). When the simulation time reaches 140, the contrast method loses accuracy and deviates from the model simulation. Conversely, as the level of the sensor fusion increases, the simulation error becomes increasingly small. On the basis of the given formula, Table 1 shows the average performance index of each level of the sensors.

From the table, the MAE of the contrast method and level-three, level-two, and level-one sensor fusion values are 10.6973, 3.5261, 3.4948, and 2.4361, respectively. The MRE of the contrast method and level-three, level-two, and level-one sensor fusion values are 1.79, 0.59, 0.58, and 0.41, respectively. The RMSE of the contrast method and level-three, level-two, and level-one sensor fusion values are 16.2528, 4.1562, 4.1297, and 3.5869, respectively. Each performance index indicates that the proposed method has a considerable improvement in measurement accuracy compared with the contrast method. In addition, the error of the simulation result may become small as the fusion level increases. Therefore, the given simulations and analysis demonstrate that the proposed algorithm can provide globally optimal fusion results. Moreover, as the level of data fusion increases, the accuracy of the proposed algorithm can be improved accordingly. This algorithm can also enhance the adaptability and robustness against linear errors to overcome the limitation of the traditional EKF.

**Figure 12:**
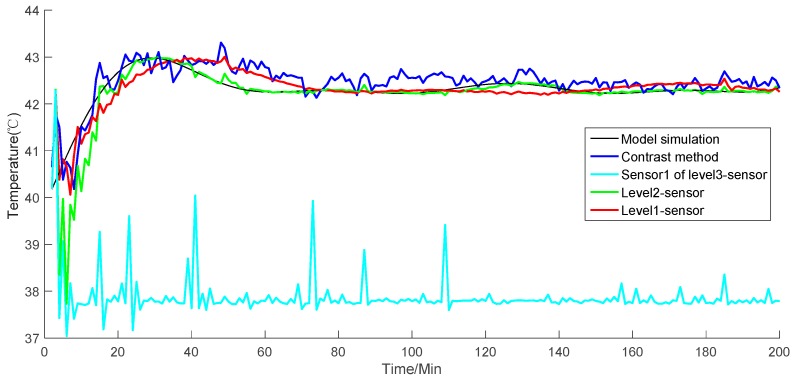
Simulation results of sensor one, level-two sensors, and level-one sensors, and the contrast method.

**Figure 13:**
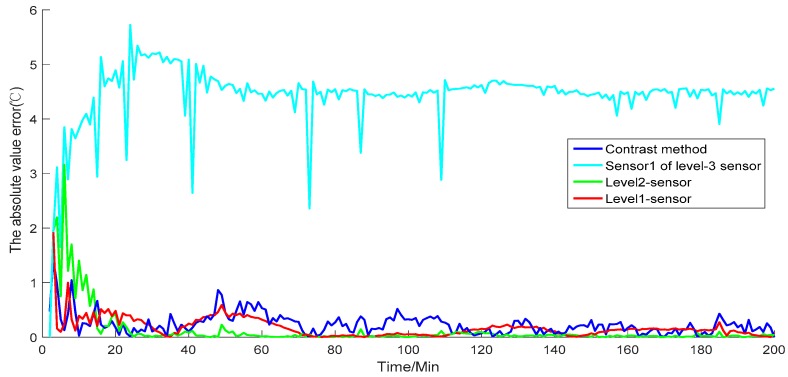
Absolute error of sensor one, level-two sensors, and level-one sensors, and the contrast method.

**Figure 14:**
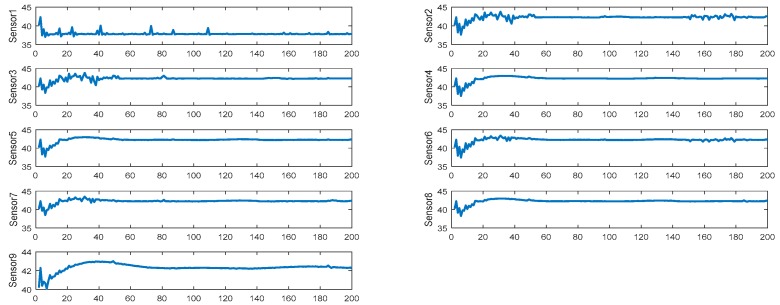
Simulation results of each sensor.

As described in Section 2.2, the fusion structure contains sensor information sharing. When a sensor is damaged, other sensors will correct the error covariance of local fusion. Dynamic weights also reduce the influence of damaged sensors, thereby reducing the effect of gross error. Consider that a level-three sensor, namely, sensor one, is damaged, and transports error data. Figure 12 shows the simulation value of temperature. Figure 13 presents the absolute value error of temperature with the damaged sensor, and Figure 14 shows each sensor value. The results in Figure 12 and Figure 13 show the simulation results and absolute value error of the damaged sensor one in level-three sensors. These values are then compared with those of the level-two and level-one sensors.

Although sensor one is damaged, the weighting principle corrects the error covariance of local fusion. The principle reduces the weight of the local fusion values, which contain damaged sensor one. Thus, high-level fusion values are affected as little as possible by the damaged sensor. As shown in Figure 12, the simulation results of the level-two local fusion in the initial stage of the simulation are not well tracked to the model simulation result due to the influence of the damaged sensor one in level three. Nevertheless, the final fusion result of level one shows that the simulation results have been well tracked to the simulated model value. The trend of the absolute value error in Figure 13 reveals that as the error of the damaged sensor increases, the results of the level-two local fusion sensors are affected at the beginning of the simulation but with the correction of the weighting criterion. The absolute value error of the final fusion value can be kept in an extremely low range. Moreover, the simulation experiment continues to achieve better results in comparison with the contrast method. Table 2 displays the average performance index of each level of the sensors. For level-three sensors, we show the performance index of sensor one.

The simulations in the two given situations show that when the temperature is stable, the proposed algorithm can fit the value smoothly when a damaged sensor exists. Level-two sensors may be affected to a certain extent in the initial stage of the simulation. Nevertheless, the results gradually improve as the simulation continues. In addition, the proposed algorithm cannot produce a large error when the temperature is stable. In comparison with the contrast method, the proposed algorithm has higher accuracy and is closer to the actual simulation value of the model; moreover, the proposed fusion algorithm has higher stability in the simulation experiment and does not produce tracking divergence. The comparison of the performance index shows that the distributed sensor fusion has higher data-filtering accuracy, smaller errors, and more accurate simulation than the contrast method based on a single sensor. The comparison of the MRE of the proposed and contrast methods indicates that the accuracy of the former is 55.39% higher than the latter.

### 3.2. Experimental Setup

**Figure 15:**
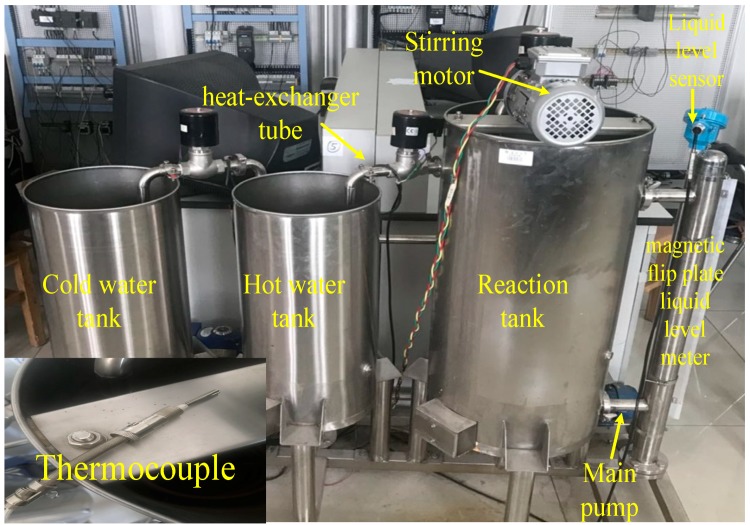
Experimental equipment diagram.

To achieve the effectiveness of distributed sensor data fusion, an experiment is set up for the biological oxidation pretreatment in the laboratory. Figure 15 shows the installation of the experiment:

As shown in Figure 15, similar to the temperature control in the actual industrial site, the installation of the experiment contains a main tank and two sub-tanks in the device. The diameter of the main tank is approximately one m, and a mixing system is installed at the top of the main tank. A snake-shaped casing is installed to regulate the temperature inside the tank. A magnetic flip-plate liquid-level meter is installed on the side, and a sensor support frame is installed inside the main box to install the temperature detection sensor. The two sub-boxes are equipped with cold and hot water, which will be used for heat exchange. The snake-shaped casing outlet is equipped with a flow sensor to detect the cold and hot water flow. Two pumps are installed at the lower end of the device to regulate the circulating water flow. These pumps also play a role in controlling the temperature. The temperature is set at 40 °C.

In the actual industrial field, the brand of sensors that we used was HACH (USA), and the type of sensor was a P53 pH/ORP sensor. This type of sensor measures the pH value, ORP value, and temperature. The temperature compensator element was a PT-1000.

In the calibration process of each sensor, a high-precision mercury thermometer was detected into the ore pulp by the workers on the spot. The temperature of the sensor position was measured and compared with the data transmitted by the sensor to check whether the sensor was inaccurate.

The high-precision mercury thermometer that was used in the industrial field was about 20–30 cm long in total and had an immersion length of 10 cm. The workers on the spot fixed the thermometer to a plastic insulation rod and only exposed the thermometer probe. In the actual industrial sites, our workers calibrated each sensor in three periods: in the morning, at noon, and in the evening. In each period, for each sensor, a group of data was measured in a short time. If it was consistent with the data transmitted by the data acquisition box, the sensor did not need to be calibrated; if there was a minor difference, the workers would calibrate each sensor using the standard electrolyte; if there was a big difference, first, the workers would check the high-precision mercury thermometer, or replace another thermometer to measure it again. If there was still a big difference, it may have been caused by the sensor. After the sensor problem was identified, we might have repaired or replaced the sensor until the sensor was consistent with the high-precision mercury thermometer. In the industrial sites, the workers would calibrate the sensors for few days, over three periods, and if they were all consistent, the sensors would be put into use. Table 3 shows the parameters of this type of sensor.

If we take into account the circumstances that were not affected by the external environment, the accuracy and precision of each sensor is the same. Table 4 shows the physical property parameters of the materials.

**Figure 16:**
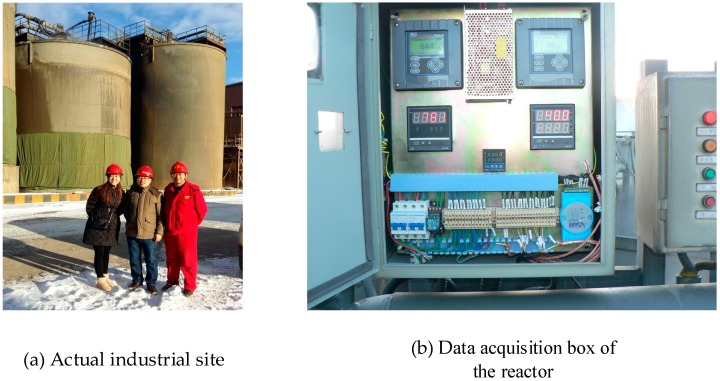
Actual industrial site and data acquisition box of the reactor.

In the actual industrial site, the measurements of each sensor could be obtained in a control box. Figure 16 shows the data acquisition box of each variable (including temperature, oxygen quantity, pH value, and potential value), and a far-away view of the reactor in the actual industrial site. 

**Figure 17:**
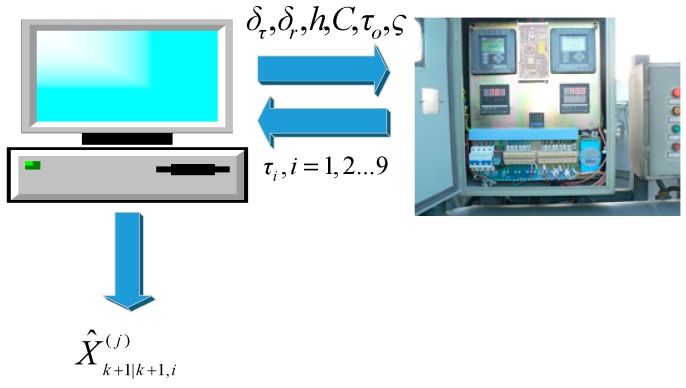
Processing flow of the experimental data.

The data acquisition of the experimental equipment was recorded on the computer by using multi-sensors to transport real-time data of the experimental temperature. When the temperature was stable in the setting value, data were recorded, and raw data were obtained. The proposed method was then used to process the raw data, and the fusion value was obtained using the original data. By comparing the deviation of the fusion value with the setting value and that of the original measurement with the setting value, we could obtain the measuring effect of the original measuring method and that of the proposed method. Figure 17 illustrates the processing flow of the experimental data.

**Figure 18:**
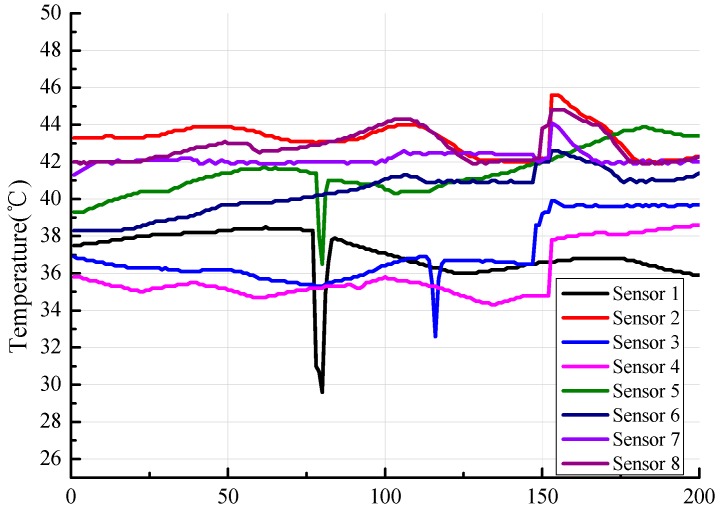
Experimental result of each sensor.

The measurement data in Section 3.2 are from the actual industrial area in Xinjiang, China during winter. We adopted 200 temperature values after stabilizing the temperature. These 200 acquisition points are discrete data, and the sample period of each point was 15 minutes. Given the different positions of the installed sensors, the measurement of each sensor may be different due to internal and external factors. Figure 18 shows the measured temperature of each sensor obtained from the experiment. The transverse coordinates represent 200 data points.

**Figure 19:**
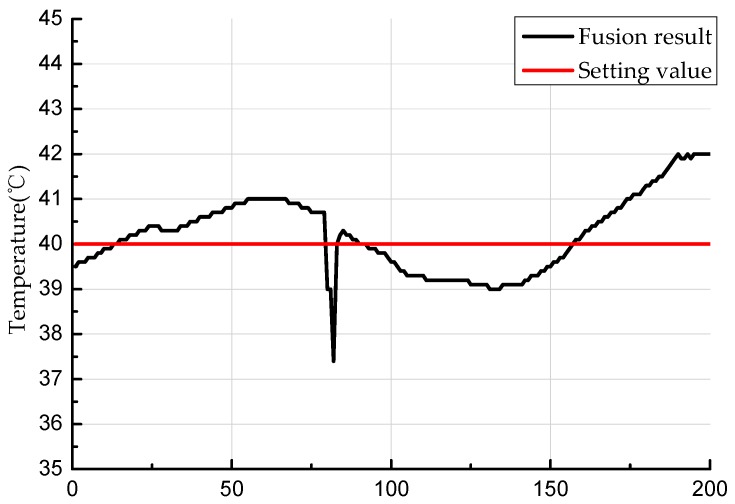
Experimental fusion result.

After obtaining the data of each sensor, the proposed algorithm was used to obtain the final experimental result. Figure 19 shows the distributed data fusion result of sensor nine.

**Figure 20:**
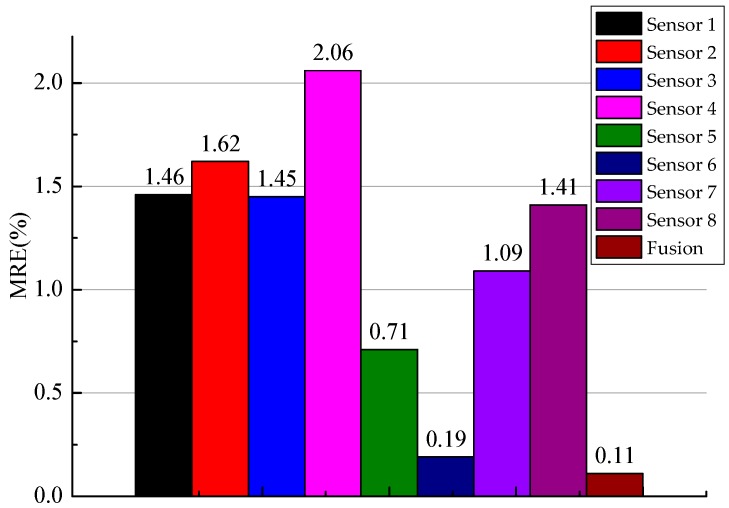
MRE of experimental result.

After obtaining all the data of each sensor and the fusion value, we could obtain the MRE of the low-level sensor and the fusion result, as shown in Figure 20.

After processing the algorithm, the accuracy of the fusion result was higher than when the algorithm was not used. The accuracy of industrial measurement improved by 36.84% when the proposed method was used in the equipment experiment.

## 4. Conclusions

This study aimed to improve the performance of temperature measurement in biological oxidation pretreatment, and thus propose a distributed sensor fusion method. The mechanism of heat transfer in biological oxidation pretreatment was analyzed, and a suitable state model was established. On the basis of the fusion structure and the industrial equipment of biological oxidation pretreatment, a small-range sensor network for practical industrial survey was set up. In data processing, the iterative operation of the state function was introduced and multi-fading factors based on weighted fading memory index were added. Iterative operation reduced the high linear errors, and the multi-fading factors adjusted the prediction error covariance, thereby reducing the accumulated error caused by the filter memory. In the fusion part, the state estimation accuracy of each sensor was used as a weighting principle to adjust the predictive confidence of each sensor by adding a dynamic weighting factor. The method was applied to the experiments of biological oxidation pretreatment, and achieved satisfactory results in the laboratory. In comparison with the traditional single-sensor state estimation, the proposed distributed state estimation schemes had higher fault tolerance.

## Figures and Tables

**Table 1:** Mean absolute error (MAE), mean relative error (MRE), and root mean square error (RMSE) of the contrast method and level-three, level-two, and level-one sensor fusion for the simulation case.

Type	Error type		
	MAE (°C)	MRE (%)	RMSE (°C)
Contrast method based on single sensor with EKF	10.6973	1.79	16.2528
Level-3 sensor fusion	3.5261	0.59	4.1562
Level-2 sensor fusion	3.4948	0.58	4.1297
Level-1 sensor fusion	2.4361	0.41	3.5869

**Table 2:** MAE, MRE, and RMSE of the contrast method, sensor one of level-three, level-two, and level-one sensors for the simulation case.

Type	Error type		
	MAE (°C)	MRE (%)	RMSE (°C)
Contrast method based on single sensor with EKF	5.4830	0.92	10.6396
Sensor one of level-three sensor	63.0346	10.56	70.2693
Level-two sensor fusion	4.2876	0.72	5.8926
Level-one sensor fusion	3.6103	0.61	4.9857

**Table 3:** Performance of sensor. ORP: oxidation-reduction potential.

Performance index	Parameter
Brand	HACH (USA) P53A4A1N
pH Range	−2.00–4.00 pH
ORP Range	−2100 ± 2100 mV
Temperature Range	−20.00 ± 200.00 °C
Temperature Compensation	−10.00 ± 110.00 °C
Accuracy	0.2% of range every 24 h, no accumulation
Precision	0.1% of range or better
Output	Two 0/4–20 mA DC RS232
Source	190–60 VAC, 50/60 Hz
Protection Degree	NEMA 4X (IP65), 1/2 DIN
Calibration	Standard electrolyte

**Table 4:** Physical property parameters of the materials.

Material	Thermal conductivity (W·m^−1^·°C^−1^)	Density (kg·m^−3^)	Specific heat (J·kg^−1^·°C^−1^)
Stainless steel	16	7900	450
Air	0.028	1.29	1030
Ore pulp	—	1150	3290
Water	0.64	1000	4186
